# A Systematic Review of the Bidirectional Relationship Between Post-traumatic Stress Disorder (PTSD) and the Development of Type 2 Diabetes

**DOI:** 10.7759/cureus.93457

**Published:** 2025-09-29

**Authors:** Naif Aleid, Ibrahim Enad Alenazi, Fahad Saleh Alghasham, Saud Abdulmajeed Bin Rakhis, Abdulraham Mansour A AlFawaz

**Affiliations:** 1 Family Medicine, King Fahad Medical City, Riyadh, SAU; 2 Primary Care Medical Administration, King Fahd Medical City, Riyadh, SAU; 3 Family Medicine, Prince Sultan Military City, Riyadh, SAU

**Keywords:** bidirectional relationship, inflammation, post-traumatic stress disorder, ptsd, stress, systematic review, trauma, type 2 diabetes

## Abstract

Emerging evidence suggests a bidirectional relationship between post-traumatic stress disorder (PTSD) and type 2 diabetes (T2D), though the underlying mechanisms and population-specific risks remain unclear. This systematic review synthesizes current research on the PTSD-T2D link, focusing on biological pathways, behavioral mediators, and demographic disparities. Following the Preferred Reporting Items for Systematic Reviews and Meta-Analyses (PRISMA) guidelines, we conducted a comprehensive search across PubMed/Medical Literature Analysis and Retrieval System Online (MEDLINE), Excerpta Medica database (Embase), PsycINFO, and Web of Science. Eligible studies examined PTSD as a risk factor for T2D or vice versa in adults (≥18 years). Two reviewers independently screened articles, extracted data, and assessed bias using the Newcastle-Ottawa Scale (NOS) and Cochrane tools. Thirteen studies met the inclusion criteria. PTSD increased T2D risk and was associated with worse glycemic control (e.g., elevated glycated hemoglobin A1c (HbA1c)). T2D populations exhibited higher PTSD prevalence (30%-50% in high-trauma groups), particularly among women, refugees, and veterans. Biological mechanisms (hypothalamic-pituitary-adrenal (HPA) axis dysregulation, chronic inflammation) and behavioral factors (sedentary lifestyle, poor adherence) drove the bidirectional relationship. Conflict-affected populations showed heightened vulnerability, with war-displaced individuals facing compounded metabolic and mental health burdens. PTSD and T2D share a complex, bidirectional relationship influenced by neuroendocrine, inflammatory, and behavioral pathways. High-risk groups (e.g., refugees, veterans, and women) may benefit from integrated screening and trauma-informed diabetes care. Future research should prioritize longitudinal designs to clarify causality and evaluate targeted interventions.

## Introduction and background

Post-traumatic stress disorder (PTSD) and type 2 diabetes (T2D) represent two major public health challenges with a growing body of evidence suggesting a bidirectional relationship between them. PTSD, a debilitating psychiatric condition triggered by exposure to traumatic events, affects approximately 3.9% of the global population, with higher prevalence rates observed in conflict-affected regions and among military veterans [1]. Concurrently, T2D has reached epidemic proportions, affecting over 537 million adults worldwide, with projections indicating a continued rise due to aging populations and lifestyle factors [2]. Emerging research indicates that individuals with PTSD are at a significantly elevated risk of developing T2D, with meta-analyses reporting a 30%-50% increased incidence compared to non-PTSD populations [3]. This association persists even after adjusting for traditional risk factors such as obesity and physical inactivity, suggesting that PTSD may exert independent metabolic effects through neuroendocrine and inflammatory pathways [4]. The biological mechanisms linking PTSD to T2D are multifaceted and involve dysregulation of the hypothalamic-pituitary-adrenal (HPA) axis, chronic low-grade inflammation, and autonomic nervous system dysfunction. Prolonged stress exposure in PTSD leads to hypercortisolism, which promotes insulin resistance and visceral fat accumulation-key drivers of T2D pathogenesis [5]. Additionally, elevated levels of pro-inflammatory cytokines (e.g., interleukin-6 (IL-6), C-reactive protein (CRP)) observed in PTSD patients contribute to β-cell dysfunction and impaired glucose metabolism [6]. Behavioral factors, including poor sleep, sedentary lifestyle, and medication non-adherence, further exacerbate this relationship, creating a vicious cycle of worsening metabolic and mental health outcomes [7]. Despite these insights, critical gaps remain in understanding how sociodemographic variables (e.g., gender, displacement status) and comorbid conditions (e.g., hypertension) modulate PTSD-T2D interactions, particularly in understudied populations such as refugees and veterans. This systematic review aims to synthesize current evidence on the bidirectional relationship between PTSD and T2D.

## Review

Methods

This systematic review was conducted in accordance with the Preferred Reporting Items for Systematic Reviews and Meta-Analyses (PRISMA) guidelines [8]. A comprehensive search strategy was implemented across multiple electronic databases, including PubMed/Medical Literature Analysis and Retrieval System Online (MEDLINE), Excerpta Medica database (Embase), PsycINFO, and Web of Science over the years 2020-2025 to identify all relevant studies examining the bidirectional relationship between PTSD and T2D. The search incorporated controlled vocabulary terms (MeSH, Emtree) and free-text keywords related to PTSD, trauma-related disorders, and T2D, with search syntax adapted for each database following recommendations by Bramer et al. [9]. To minimize selection bias, two independent reviewers performed study screening, selection, data extraction, and quality assessment using standardized protocols. Discrepancies were resolved through consensus or consultation with a third reviewer when necessary. To ensure the non-redundant inclusion of data, studies originating from the same cohort or dataset were carefully examined. When overlaps were identified, the publication with the most comprehensive data or the longest follow-up period was selected for inclusion.

Eligibility Criteria

Studies were included if they (1) investigated either the risk of T2D development in PTSD populations or the risk of PTSD development in T2D populations; (2) included adult participants (≥18 years); (3) provided quantitative data on PTSD diagnosis (clinical or validated scales) and T2D outcomes (incidence, prevalence, or metabolic markers); and (4) were published in English. We excluded studies focusing exclusively on type 1 diabetes, gestational diabetes, or prediabetes; those without primary data (reviews, editorials); animal studies; and case reports with fewer than 10 participants. Both observational (cohort, case-control, and cross-sectional) and interventional studies were eligible.

Data Extraction

A piloted, standardized form was used to extract data on study design, sample characteristics (demographics, PTSD/T2D diagnostic criteria), key findings (effect sizes, adjusted odds ratios (ORs)/hazard ratios), and confounding variables. For studies reporting longitudinal outcomes, we extracted data on follow-up duration and attrition rates. Reference management software (EndNote X9, Clarivate, London, UK) and the Rayyan platform (Rayyan Systems Inc., Cambridge, MA) [10] were employed to streamline screening and eliminate duplicates. All included studies underwent full-text review, with data extraction performed independently by two researchers to ensure accuracy.

Data Synthesis Strategy

Given the heterogeneity in study designs and outcomes, a narrative synthesis was prioritized. Data were organized thematically to address (1) PTSD as a risk factor for T2D, (2) T2D as a risk factor for PTSD, and (3) mediating mechanisms (e.g., inflammation, HPA axis dysfunction). Summary tables were generated to compare effect sizes, adjustment for confounders, and population-specific findings. Where feasible, quantitative synthesis (e.g., meta-analysis of ORs) was performed for studies with comparable methodologies.

Risk of Bias Assessment

Study quality was evaluated using the Newcastle-Ottawa Scale (NOS) for observational studies [11] and the Cochrane Risk of Bias Tool for randomized trials [12]. The NOS assessed selection (representativeness, exposure/outcome ascertainment), comparability (control for confounders), and outcome/exposure assessment. For genetic studies (e.g., Mendelian randomization), we applied the Strengthening the Reporting of Observational Studies in Epidemiology using Mendelian Randomization (STROBE-MR) checklist [13]. Studies were categorized as low, moderate, or high risk based on predefined thresholds.

Results

**Figure 1:**
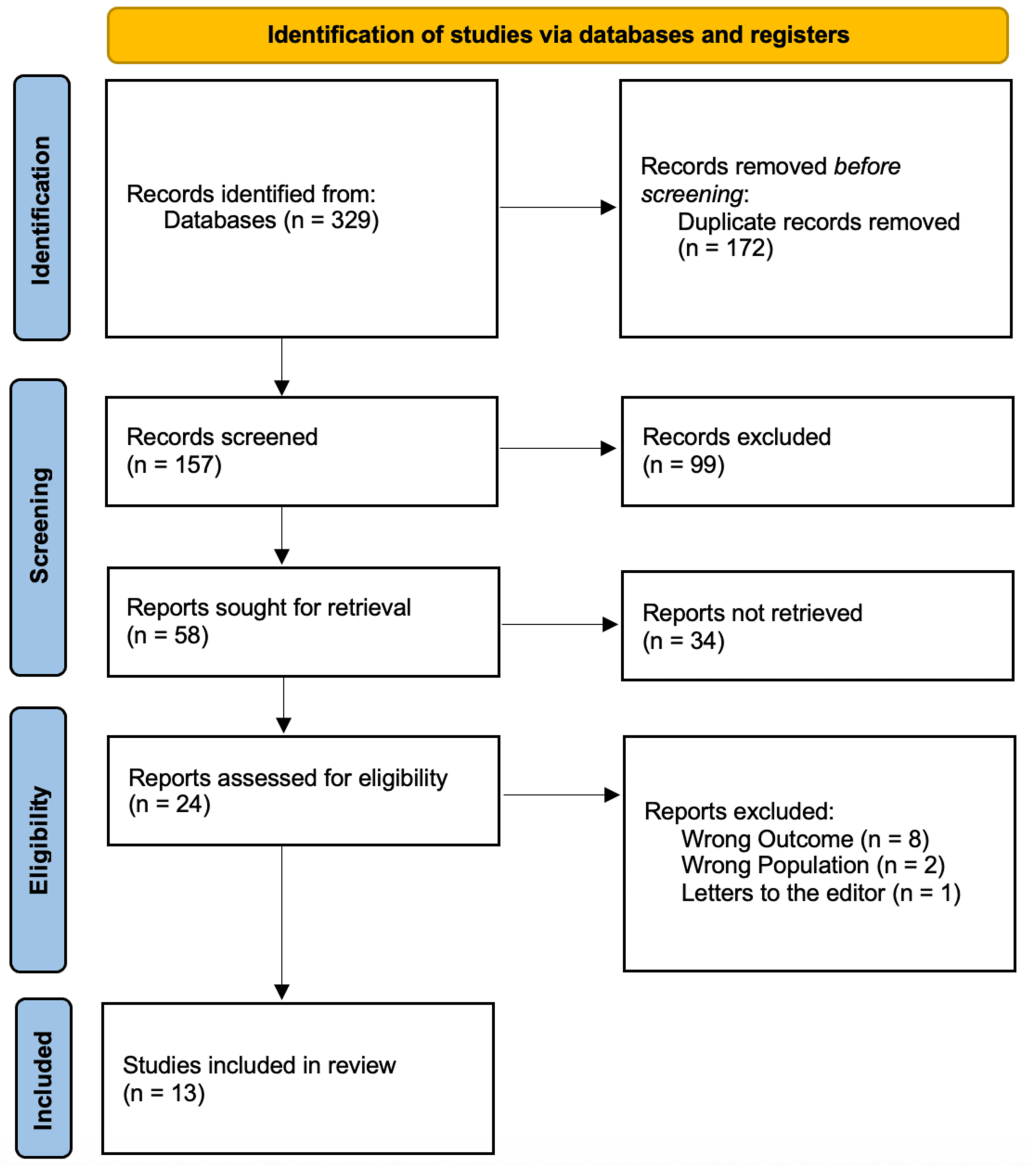
PRISMA Flow Diagram of the Study Selection Process PRISMA: Preferred Reporting Items for Systematic Reviews and Meta-Analyses

Figure 1 presents a PRISMA flow diagram outlining the systematic study selection process. Initially, 329 records were identified through database searches, with 172 duplicates removed, leaving 157 records for screening. After title/abstract screening, 99 records were excluded, and 58 full-text articles were sought for retrieval. Of these, 34 could not be retrieved, leaving 24 reports assessed for eligibility. After full-text review, 11 reports were excluded (eight for wrong outcomes, two for wrong population, and one for being an abstract), resulting in 13 studies meeting all inclusion criteria for the final review.

Table 1 summarizes demographic and study characteristics, providing a structured overview of research design, population, and sample attributes [14-26]. For instance, Gammoh et al. (2025) [14] conducted a cross-sectional study on Syrian refugees in Jordan, revealing that 69.8% of participants with hypertension or T2D exhibited severe PTSD symptoms. Similarly, Dixon et al. (2020) [17] focused on low-income African-American women with diabetes, finding that 30.4% met PTSD criteria, with trauma exposure significantly worsening glycemic control. Military and disaster-affected populations were also well-represented, such as in Hirai et al. (2022) [18], which linked PTSD to incident T2D in male Fukushima evacuees, and Bergman et al. (2022) [20], where Scottish veterans with PTSD had a 29% higher T2D risk than non-veterans.

**Table 1:** Demographics and Characteristics of the Selected Studies HTN: hypertension; T2DM: type 2 diabetes mellitus; PTSD: post-traumatic stress disorder; NM: not mentioned; T1D: type 1 diabetes; IDP: internally displaced persons; RTC: residents of temporarily occupied territories; SF-36: 36-Item Short Form Survey; HADS: Hospital Anxiety and Depression Scale; CHD: coronary heart disease; IDD: insulin-dependent diabetes; APS-SF: Adolescent PTSD Scale – Short Form.

Study (Author, Year, Reference)	Country	Study Design	Sample Size	Population Characteristics	Age (Mean/%)	Gender (% Female)	Key Demographic Notes
Gammoh et al. (2025) [14]	Jordan	Cross-sectional	327	Syrian refugees with HTN/T2DM	>50 years: 56.7%	50.3%	War-displaced, high PTSD prevalence
Arigo et al. (2020) [15]	USA	Observational	184	Adults with poorly controlled T2DM	NM	NM	No psychiatric history
Yu et al. (2024) [16]	Multi-country	Mendelian randomization	NM	Genetic datasets (GWAS)	NM	NM	Focus on obesity mediation
Dixon et al. (2020) [17]	USA	Cross-sectional	290	Low-income African-American women with T1D/T2D	NM	100%	High trauma/PTSD rates
Hirai et al. (2022) [18]	Japan	Longitudinal	NM	Fukushima disaster evacuees	NM	NM (men analyzed)	PTSD linked to incident T2D in men
Serhiyenko et al. (2025) [19]	Ukraine	Cross-sectional	64 (32 IDP)	T2DM patients vs. IDP with T2DM	NM	NM	Validated distress tool
Bergman et al. (2022) [20]	Scotland	Retrospective cohort	78,000 veterans	UK military veterans	NM	NM	PTSD-T2D comorbidity
Mykytyuk et al. (2024) [21]	Ukraine	Cross-sectional	91 (26 T1D, 65 T2D)	IDP/RTC in war zones	T1D: 34.7±8.79; T2D: 56.5±10.79	NM	SF-36/HADS used
Venkatachalam et al. (2023) [22]	Lebanon	Cross-sectional	NM	Syrian refugee women	35–55 years	100% (subset)	High inflammation/T2D
Popenko et al. (2022) [23]	Ukraine	Case-control	106 (61 ATO veterans)	Military personnel with T2DM	30–70 years	0% (male-only)	PTSD in 42.6%
Koval et al. (2023) [24]	Ukraine	Cross-sectional	56	Civilians with HTN/T2DM in war zones	NM	NM	HADS/Spielberger scales
Serik et al. (2023) [25]	Ukraine	Observational	106	Civilians with CHD/T2DM	NM	NM	PCL-5/GAD-7 used
Lica et al. (2021) [26]	Romania	Cross-sectional	54 (IDD adolescents)	Adolescents with IDD	12–18 years	NM	APS-SF tool

Table 2 synthesizes key findings, emphasizing PTSD measurement tools, T2D outcomes, and mediating factors. Studies like Yu et al. (2024) [16] used Mendelian randomization to demonstrate that PTSD increases T2D risk (OR 1.036), with obesity mediating 9.51% of this effect. Clinical studies, such as Arigo et al. (2020) [15], highlighted that PTSD symptoms in adults with poorly controlled T2D were associated with elevated diabetes distress (R² ~3%), independent of depression. War-related studies, including Venkatachalam et al. (2023) [22] and Popenko et al. (2022) [23], identified higher PTSD prevalence in conflict zones, correlating with worse metabolic outcomes (e.g., HbA1c 12.1 mmol/L in Ukrainian veterans). Gender disparities were notable, with women refugees showing higher PTSD-related inflammation [22] and civilians in war zones exhibiting severe anxiety-depression comorbidity [24].

**Table 2:** Key Findings and Variables PTSD: post-traumatic stress disorder; T2D: type 2 diabetes; HTN: hypertension; NM: not mentioned; GWAS: genome-wide association study; OR: odds ratio; HR: hazard ratio; PSS: PTSD Symptom Scale; K6: Kessler Psychological Distress Scale; PCL-S: PTSD Checklist – Specific; T2-DDAS: Type 2 Diabetes Distress Assessment Scale; IDP: internally displaced persons; HADS: Hospital Anxiety and Depression Scale; SF-36: 36-Item Short Form Survey; DBP: diastolic blood pressure; HTQ: Harvard Trauma Questionnaire; HSCL-25: Hopkins Symptom Checklist-25; SAA: serum amyloid A; ATO: anti-terrorist operation; CHD: coronary heart disease; PCL-5: PTSD Checklist for Diagnostic and Statistical Manual of Mental Disorders, Fifth Edition (DSM-5); GAD-7: Generalized Anxiety Disorder 7-Item Scale; IDD: insulin-dependent diabetes; APS-SF: Adolescent PTSD Scale – Short Form

Study (Author, Year, Reference)	PTSD Measure	T2D Measure	Key Findings	Mediators/Confounders
Gammoh et al. (2025) [14]	Arabic PTSD scale	Self-reported HTN/T2DM	69.8% had severe PTSD linked to medication lack/chronic pain	NM
Arigo et al. (2020) [15]	Self-reported symptoms	HbA1c (9.13%)	PTSD symptoms associated with diabetes distress (R² ~3%)	Depression
Yu et al. (2024) [16]	GWAS data	Genetic T2D risk	PTSD increased T2D risk (OR 1.036); mediated by obesity (9.51%)	Obesity, hypertension
Dixon et al. (2020) [17]	PSS	HbA1c	PTSD associated with higher HbA1c (p=0.002)	Childhood trauma
Hirai et al. (2022) [18]	K6/PCL-S	Incident T2D	PTSD/depression predicted T2D in men (HR sig.)	Disaster-related stress
Serhiyenko et al. (2025) [19]	T2-DDAS	HbA1c	IDP had higher distress (hypoglycemia, healthcare access)	NM
Bergman et al. (2022) [20]	Clinical records	T2D diagnosis	Veterans with PTSD had higher T2D (OR 1.29)	Mood disorders
Mykytyuk et al. (2024) [21]	HADS/SF-36	HbA1c/DBP	Warzone residents had worse mental health (p<0.05)	Anxiety/depression
Venkatachalam et al. (2023) [22]	HTQ/HSCL-25	Undiagnosed T2D	Women had higher PTSD/inflammation (SAA: p=0.036)	Obesity
Popenko et al. (2022) [23]	Clinical PTSD	HbA1c (12.1 mmol/L)	ATO veterans had higher HbA1c vs. controls (7.63 mmol/L, p<0.05)	PTSD (42.6%)
Koval et al. (2023) [24]	HADS/Spielberger	NM	80% anxiety, 69% depression; women were more affected	HTN severity
Serik et al. (2023) [25]	PCL-5/GAD-7	HbA1c/lipids	CHD/T2DM patients had higher PTSD scores (p=0.037)	Visceral fat
Lica et al. (2021) [26]	APS-SF	HbA1c (>7.6)	Girls with IDD had higher anxiety/PTSD (p<0.05)	Poor adherence

Table 3 shows that most studies demonstrated low-to-moderate risk, with Mendelian randomization [16] and registry-based designs [20] having the lowest bias due to robust methodologies. Cross-sectional studies [14, 19, 21-22] faced a moderate risk from self-reported outcomes or convenience sampling. Military studies [20, 23] had higher bias due to selection (e.g., veterans only) or unmeasured confounders (e.g., combat exposure severity). Tools like the PTSD Checklist for Diagnostic and Statistical Manual of Mental Disorders, Fifth Edition (DSM-5) (PCL-5) [25] and Harvard Trauma Questionnaire (HTQ) [22] improved reliability in PTSD measurement, whereas self-reported glycated hemoglobin A1c (HbA1c) [14, 23] introduced variability. Overall, longitudinal designs [18] and genetic analyses [16] were most rigorous, while warzone studies [21, 24-25] struggled with uncontrolled environmental stressors.

**Table 3:** Risk of Bias Assessment GWAS: genome-wide association study; MR: Mendelian Randomization; PSS: PTSD Symptom Scale; BDI: Beck Depression Inventory; K6: Kessler Psychological Distress Scale; PCL-S: PTSD Checklist – Specific; T2-DDAS: Type 2 Diabetes Distress Assessment Scale; NM: not mentioned; SF-36: 36-Item Short Form Survey; HADS: Hospital Anxiety and Depression Scale; HTQ: Harvard Trauma Questionnaire; HSCL-25: Hopkins Symptom Checklist-25; ATO: Anti-Terrorist Operation; CHD: coronary heart disease; PCL-5: PTSD Checklist for DSM-5; GAD-7: Generalized Anxiety Disorder 7-Item Scale; APS-SF: Adolescent PTSD Scale – Short Form

Study (Author, Year, Reference)	Risk of Bias Tool	Selection Bias	Comparability	Outcome/Exposure Measurement	Overall Risk
Gammoh et al. (2025) [14]	NOS	Low (representative sample)	Moderate (adjusted for pain/medications)	High (self-reported PTSD)	Moderate
Arigo et al. (2020) [15]	NOS	Low (defined T2D cohort)	High (no psychiatric history)	Low (validated scales)	Moderate
Yu et al. (2024) [16]	STROBE-MR	Low (GWAS data)	Low (MR controls)	Low (robust sensitivity tests)	Low
Dixon et al. (2020) [17]	NOS	Moderate (clinic-based)	Moderate (adjusted for trauma)	Low (PSS/BDI)	Moderate
Hirai et al. (2022) [18]	NOS	Low (longitudinal)	Low (multivariate adjustment)	Low (K6/PCL-S)	Low
Serhiyenko et al. (2025) [19]	NOS	Moderate (small sample)	NM	Moderate (T2-DDAS validation)	Moderate
Bergman et al. (2022) [20]	NOS	Low (national registry)	Low (matched controls)	Low (clinical records)	Low
Mykytyuk et al. (2024) [21]	NOS	Moderate (convenience sample)	NM	Moderate (SF-36/HADS)	Moderate
Venkatachalam et al. (2023) [22]	NOS	Moderate (refugee subset)	Moderate (adjusted for sex/age)	Low (HTQ/HSCL-25)	Moderate
Popenko et al. (2022) [23]	NOS	High (military bias)	Moderate (case-control)	High (self-reported PTSD)	High
Koval et al. (2023) [24]	NOS	Moderate (warzone sample)	NM	Moderate (HADS/Spielberger)	Moderate
Serik et al. (2023) [25]	NOS	Moderate (CHD/T2DM subset)	Low (adjusted for lipids)	Low (PCL-5/GAD-7)	Moderate
Lica et al. (2021) [26]	NOS	Low (adolescent cohort)	NM	Moderate (APS-SF)	Moderate

Discussion

This review synthesizes evidence on the complex relationship between PTSD and T2D, corroborating and expanding upon existing literature that highlights a significant association between the two conditions. Our analysis underscores critical nuances involving risk factors, mediating mechanisms, and population-specific vulnerabilities.

The aggregated findings indicate a robust association between PTSD and an increased risk for T2D, with obesity and hypertension emerging as key mediating factors [16, 27]. For instance, genetic evidence from a Mendelian randomization study by Yu et al. (2024) supports a potential causal pathway, demonstrating that genetic liability to PTSD is associated with a higher risk of T2D, with obesity mediating a significant portion of this effect [16]. This aligns with longitudinal data, such as from the Nurses’ Health Study II, which reported a 1.5- to two-fold increased incidence of T2D in women with PTSD [28]. Beyond behavioral mediators, our review identified chronic stress and inflammation as exacerbating factors. This is consistent with research in veteran populations, where inflammatory markers like CRP and IL-6 have been shown to mediate the PTSD-T2D relationship [29].

The context of trauma and demographic factors appear to be pivotal modifiers of risk. Disparities are particularly evident by gender. While one study of disaster survivors found PTSD was predictive of T2D only in men [18], other research involving refugees and trauma-exposed civilians has documented a disproportionately high PTSD prevalence and worse glycemic control among women [17, 22]. This observation aligns with meta-analytic data suggesting women with PTSD may face a greater T2D risk than men, possibly due to sex-specific hormonal interactions [30, 31]. Military and conflict-affected populations also exhibit unique vulnerabilities. Studies consistently show that veterans with PTSD have a higher incidence of T2D compared to their civilian counterparts [20, 32]. Furthermore, research in war zones, such as studies on Ukrainian veterans and internally displaced persons (IDPs), highlights the compounding effects of chronic stress, with these groups displaying significantly higher diabetes distress and worse metabolic outcomes [19, 21, 23].

The pathophysiological pathways linking PTSD to T2D likely involve dysregulation of the HPA axis, leading to increased cortisol secretion and subsequent insulin resistance, as reflected in studies showing elevated fasting glucose in individuals with PTSD [17, 25, 33, 34]. Concurrently, PTSD-associated pro-inflammatory cytokines (e.g., tumor necrosis factor-alpha (TNF-α), IL-6) may further impair β-cell function [22, 29]. These biological mechanisms are often compounded by behavioral sequelae of PTSD, such as physical inactivity, poor diet, and medication non-adherence, which collectively worsen metabolic health [23, 26].

From a clinical perspective, these findings underscore the necessity for integrated screening and management strategies that address both PTSD and T2D, particularly in high-risk groups such as refugees, veterans, and survivors of chronic trauma. The validation of targeted tools, like the Ukrainian Type 2 Diabetes Distress Assessment System (T2-DDAS) for diabetes distress, demonstrates their utility in identifying at-risk individuals within specific populations [19]. Moreover, the evidence supporting the mediating role of obesity suggests that weight management and metabolic health interventions could be valuable components of a comprehensive treatment approach for individuals with PTSD [16].

Limitations

This review has several limitations that should be considered when interpreting the findings. First, the significant heterogeneity in how the key variables were measured poses a challenge for direct comparison across studies. PTSD was assessed using a range of tools, from clinical interviews and standardized scales like the PCL-5 to simpler self-report measures [14, 23]. Similarly, T2D was defined inconsistently, using criteria from HbA1c levels to International Classification of Diseases (ICD)-coded clinical diagnoses [17, 20]. Second, the overreliance on cross-sectional data, particularly in studies of conflict-affected populations [21, 24, 25], prevents the determination of temporal sequence and the establishment of causal inferences between PTSD and T2D. A further critical limitation is the imbalance in the evidence base. Many studies, especially those conducted in warzone settings, had small sample sizes (e.g., <100 participants), yet their findings are often reported with weights comparable to large-scale registry or genetic studies; this may inadvertently overstate preliminary or weak evidence. Additionally, the generalizability of the findings is constrained by demographic and geographic biases. For instance, the genetic evidence from Mendelian randomization studies is primarily based on European genome-wide association study (GWAS) data, limiting its applicability to diverse, non-European populations [16]. Furthermore, research focused on military and veteran cohorts [20, 23] often lacks adequate representation of female participants, which may obscure important sex-specific mechanisms and risk profiles.

## Conclusions

PTSD elevates T2D risk through biological (inflammation, HPA dysfunction) and behavioral pathways, with heightened vulnerability in women, refugees, and veterans. Clinicians should prioritize trauma-informed diabetes care, including routine PTSD screening in T2D populations and vice versa. Future research must employ longitudinal designs in diverse cohorts to clarify causal mechanisms and evaluate targeted interventions (e.g., anti-inflammatory therapies, stress-reduction programs).
